# Updating Perspectives on Meta-Analyses in the Field of Radiation Oncology

**DOI:** 10.3390/medicina57020117

**Published:** 2021-01-28

**Authors:** In-Soo Shin, Chai Hong Rim

**Affiliations:** 1 Department of Transdisciplinary Security, Dongguk University, Seoul 04620, Korea; s9065031@dongguk.edu; 2 Department of Radiation Oncology, Ansan Hospital, Korea University Medical College, Ansan-si 15355, Korea

**Keywords:** radiation oncology, meta-analysis, systematic review, observational studies, radiotherapy

## Abstract

Meta-analyses have been conventionally performed to extract the firmest conclusions from randomized controlled trials while minimizing the risk of bias. However, the field of oncology does not always allow for collecting the best evidence. Radiation oncology is a discipline where intractable or rare diseases are commonly encountered; hence, more practical data suitable for detailed clinical evaluations are needed. This review discusses new viewpoints regarding meta-analyses by pointing out heterogeneities among clinical studies and issues related to analyzing observational studies, thus clarifying the practical utility of meta-analyses in radiation oncology. Limitations of previous systematic reviews or meta-analyses are also assessed to suggest future directions.

## 1. Introduction

Meta-analyses are advantageous because they integrate multiple results to obtain a representative value that is more applicable in clinical practice. The impact of the evidence can be enhanced by overcoming the limitations of a single study. Heterogeneity among studies can also be assessed quantitatively, which allows for clinical interpretation. So far, meta-analyses in medicine have been classically used to establish the firmest possible evidence by integrating data from randomized controlled trials (RCTs) [1], thereby minimizing the risk of any bias.

Cancer is the second leading cause of death globally [2]; it is also the primary cause of death in East Asia [3,4,5], where relatively few deaths from cardiovascular disease occur. Oncology is one of the most actively studied fields in medicine and also a major field of collaboration among a variety of medical disciplines.

Approximately, 60% of all patients with cancer are referred to radiation oncology departments to undergo radiotherapy [6]. However, most patients are primarily diagnosed or treated within other disciplines such as internal medicine or surgery before being referred. In addition, radiotherapy often competes with other curative treatments and is unlikely to remain the primary treatment modality. Hence, from a research point of view, it is relatively difficult to conduct prospective studies with efficient patient selection. Patients who have intractable diseases with heterogeneous clinical characteristics are commonly referred to undergo radiotherapy and are therefore less suitable for inclusion in clinical trials. Investor-led research might also be difficult to conduct because radiation oncology does not provide sustained economic benefits to vendors through the continued use of drugs or other medical supplies. For the abovementioned reasons, many radiation oncology studies are observational and retrospective in nature and are therefore criticized for having low levels of evidence.

Nonetheless, radiation oncologists commonly encounter patients who are refractory or are difficult to manage with standardized primary treatments. As stated by the National Comprehensive Cancer Network [7], the firmest evidence in the field of oncology is not always available from RCTs, and clinical decisions are commonly dependent on non-randomized or early-phase trials, multiple retrospective studies, or even clinical expertise. In the same vein, radiation oncology is a discipline in which physicians commonly face patients with intractable diseases with limited information that is based on observational or retrospective studies. Therefore, the interpretation and application of meta-analyses within the field of radiation oncology might require some different approaches.

## 2. Utility of Meta-Analyses in the Field of Radiation Oncology

**Figure 1:**
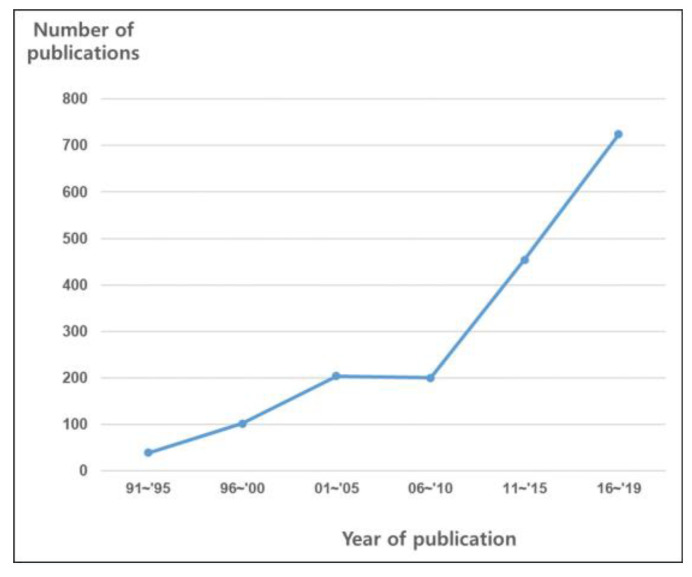
Graph showing the crude numbers of full-text meta-analyses of *radiation oncology* (based on searches of EMBASE and MEDLINE).

Nowadays, radiotherapy and/or chemotherapy are considered the standard primary modality for many types of cancer. Application of radiotherapy for some cancers is based on well-designed RCTs, such as those researching cervical cancer [8]. For some cancers, clinical experiences and related studies have enabled the acceptance of radiotherapy as a standard primary modality. Clinical data accumulated since the 1970s [9,10], when anal cancers began to be treated using chemoradiation without the use of a stoma, established concurrent chemoradiation as the primary intervention [11]. Radiotherapy and/or chemotherapy have produced similar oncologic outcomes to those of surgery for head and neck cancer [12,13] and prostate cancer [14,15]; the treatment modality is selectively applied depending on the physician’s discretion, clinical situation, or patient’s preference. The number of published meta-analyses has also rapidly increased following the broadening of indications for these modalities (Figure 1).

While there are some diseases (including the abovementioned) for which the role of radiation therapy is established, its effectiveness against other malignancies remains controversial. Treating early lung cancer with stereotactic body radiotherapy (SBRT) has attracted attention in recent decades owing to its curative role. Although RCTs comparing SBRT and surgery have shown promising results with SBRT, criticisms including poor accrual and imperfect study designs are difficult to avoid [16]; hence, SBRT is commonly indicated only for inoperable cases [17]. The prevalence of liver cancer is overwhelmingly higher in East Asia than in Europe [5], and based on their abundant clinical experiences, researchers in East Asia have found radiation therapy to be effective against intractable liver cancer [18,19,20]. However, some international guidelines still do not recognize radiotherapy as a standard intervention, mainly because of the low level of evidence given the lack of properly designed RCTs [21].

**Figure 2:**
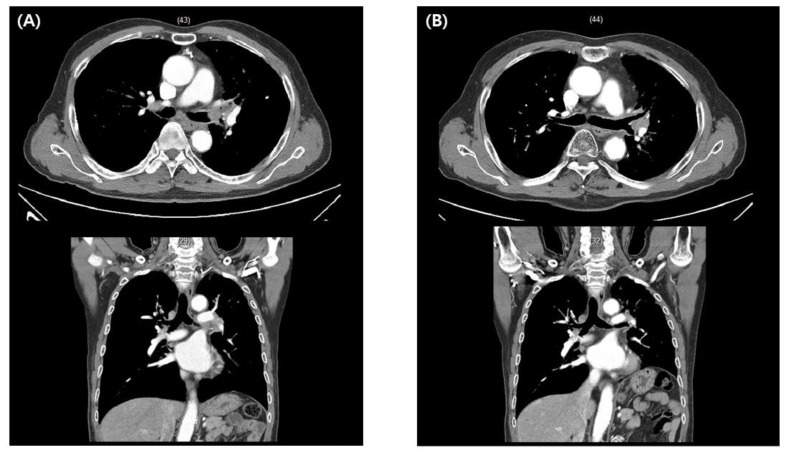
A 70-year-old man diagnosed with non-small cell lung cancer involving the left main bronchus (**A**). Stereotactic body radiotherapy with moderate intensity (50 Gy/10 F) was performed after he refused pneumonectomy, as was initially recommended. The patient is alive without evidence of disease one and a half years after treatment completion (**B**).

Let us review the following two clinical cases to illustrate the clinical efficacy of meta-analyses. The first patient was a 70-year-old man who was diagnosed with non-small cell lung cancer that invaded the left main bronchus. The primary treatment recommended after diagnosis was pneumonectomy; however, the patient strongly refused the surgery, and a multidisciplinary meeting was held to decide the next course of action. Although none of the guidelines clearly dictated the application of radiotherapy in this case, we opted for SBRT based on a clinical case series that provided evidence of its potential curability [22]. The patient has been disease-free for one and a half years after SBRT with no significant toxicity reported (Figure 2).

**Figure 3:**
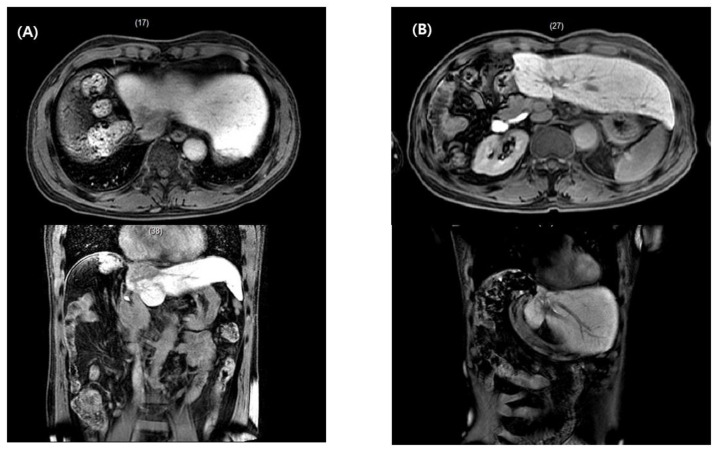
A 65-year-old man experienced a recurrent hepatocellular carcinoma lesion that reached from the inferior vena cava to the right atrium after prior right hepatectomy (**A**). After conformal radiotherapy followed by trans-arterial chemoembolization and systemic treatment, the patient is alive and disease-free seven years post-radiotherapy completion (**B**).

The second patient experienced liver cancer recurrence at the inferior vena cava and right atrium after a previous hepatectomy. Based on data from previous RCTs, the only intervention in these clinical situations is systemic treatment using sorafenib, which has a tumor response rate of 2–3% [23,24]. After multidisciplinary discussions, however, we performed conformal radiotherapy and embolization on the recurrent tumors followed by systemic treatment. The patient has been disease-free for seven years since treatment (Figure 3).

When treating “beyond the textbook,” as in the above examples, clinicians manually refer to as many studies as possible while making treatment decisions. In other words, oncologists in practice often “meta-analyze” studies in their own way. Such medical decisions can be made more efficiently and accurately based on formal meta-analysis results. In the first patient above, radiation oncologists can suggest the usefulness of SBRT for a bronchus-abutting tumor based on a recent meta-analysis that demonstrated a pooled two-year local control rate of 96.7% (95% confidence interval (CI): 91–99%). The non-negligible complication rate (i.e., pooled grade 3 complication rate) of 23.2% (95% CI: 11.8–40.5) indicates that physicians must carefully consider possible risk factors such as squamous histology and bevacizumab or anticoagulant use [22]. Although the second patient had a very rare disease known to have an extremely poor prognosis, radiotherapy could be considered in such a case based on the results of a recent meta-analysis showing that the tumor response and one-year survival rates were 59.2% (95% CI: 39.0–76.7%) and 53.6% (95% CI: 45.7–61.3%), respectively [19].

In summary, meta-analyses of clinical studies, even if they are not RCTs, can provide more helpful information than individual reports and might contribute to the successful application of radiotherapy in previously unexplored areas. The simple, integrated results of meta-analyses can be useful in multidisciplinary practices that are increasingly being applied in clinical practice. In addition, future research can be more effectively designed based on the meta-analysis effect size rather than on casual reviews of the literature.

## 3. Heterogeneity and the Effects Model

In the field of therapeutics, systematic reviews and meta-analyses depend more on RCTs than on epidemiology or diagnostics. Only 10% of the meta-analyses involving epidemiology and diagnostics were based on RCTs, whereas nearly 90% of those involving therapeutics were based on the same [25]. In a review that focused on the field of radiation oncology, 64% of the meta-analyses were based on RCTs, which was a lower proportion than that for therapeutics. In addition, the most common reason that formal meta-analyses were not performed as part of systematic reviews was the heterogeneity between the studies [26].

Heterogeneity between studies is inevitable when performing a clinical meta-analysis, as such studies integrate information from institutions with different treatment schemes and patient characteristics. However, heterogeneity among studies is not an obstacle for conducting meta-analyses; rather, it is a subject that should be rigorously interpreted and clinically analyzed with statistical analysis, such as subgroup comparison or meta-regression [27]. Meta-analyses of radiation oncology studies in particular require an understanding and interpretation of heterogeneity.

Let us take another example of treating liver cancer, for which radiotherapy is not yet recognized as a standard intervention. One of the most well-known studies of liver cancer treatment since the 2000s is the SHARP trial published in the *New England Journal of Medicine* in 2008 [23]. This study was the first RCT to demonstrate a survival benefit with chemotherapy in patients with liver cancer. The study, led by internal hepatologists, was a well-designed RCT that prospectively evaluated a large number of patients (selected according to strict inclusion criteria) from 121 centers in 21 countries and was well-supported by vendors.

On the other hand, radiation oncologists often encounter patients who are unable to undergo treatment with the standard primary modality in medicine or surgery. In the field of radiation oncology, one of the most well-studied areas in relation to liver cancer is portal vein thrombosis [28], for which standardized primary treatments such as surgery and embolization are not easily indicated. Although numerous publications exist, almost all studies were retrospective and/or observational and were based on clinical experiences of individual affiliations [18].

Physicians who primarily treat large numbers of patients can identify the appropriate cases for inclusion in prospective, well-designed studies. However, radiation oncologists commonly encounter referred patients with intractable diseases and heterogeneous or unspecified clinical conditions. Relatively limited human resources and vendor support are other hindrances to designing high-level studies with fewer biases and less heterogeneity [29,30].

Regarding meta-analyses of studies with significant heterogeneity, “comparing apples to oranges” is a common metaphoric criticism. Robert Rosenthal’s response “It makes sense if your goal is to produce fruit salad” might be a fitting description for meta-analyses in radiation oncology [31]. For example, if we conducted a meta-analysis using very strict criteria, including prospective controlled trials with little heterogeneity in study design and patients, it can be assumed that a pooled effect size with a very narrow confidence interval will be obtained—but does this result help solve clinical challenges in the real world, especially in such fields commonly encountering intractable and heterogeneous cases? Most clinicians would probably know or assume the answers to the study questions posed without resorting to a meta-analysis. Heterogeneity among studies in meta-analyses is a subject to be statistically assessed and clinically interpreted, rather than being regarded as an obstacle for drawing conclusions. In other words, the purpose of a good meta-analysis is to *synthesize* rather than simply report pooled effect sizes, and to explain phenomena while helping to direct clinical decisions, thereby sharing the same purpose as primary studies [32].

Effects that are constant indicate that the studies included in the analysis have similar designs and clinical features and also that the results of the effect size is robust. However, the presence of modest or substantial dispersion does not indicate that the analysis is useless. Rather, it indicates that the dispersion itself, as well as the summary effect, should be discussed and its cause explored through further analyses such as subgroup comparisons (categorical analysis) or meta-regression (continuous analysis) [31].

To discuss heterogeneity among radiation oncology studies included in meta-analyses, the necessary statistical concepts will be briefly explained herein. An advantage of meta-analyses is that, unlike narrative reviews, they quantitatively identify heterogeneity among studies. Although there are several parameters indicating heterogeneity, the most commonly and practically used are the *p*-values of Cochran’s Q statistic [33] and I^2^ values [34]. The former is a test of the null hypothesis Q that all studies have a common effect size; the *p*-value is used to test this null hypothesis, not to assess the degree of dispersion, because the value is affected by the number of studies analyzed. The I^2^ value ranges from 0% to 100% and conceptually reflects the proportion of variance between studies divided by total variance; this value should be used as a measure of inconsistency and not as a tool to verify the actual variation between true effects.

When calculating pooled effect sizes through meta-analyses, either fixed or random effects models should be selected. The fixed model is based on the assumption that all studies included in the analysis are functionally identical and that there is a common effect size, with any differences in the observed effects caused by sampling error [35]. The random effects model assumes that the true effect size varies among studies and is used to estimate the mean distribution of the effects. Hence, the variance in the pooled effect size is much more dependent on the sample size in each study when using the fixed effects model. Some researchers choose analysis models by determining the significance of the homogeneity among studies based on *p*-values or I^2^ values. This approach is strongly discouraged, as Borenstein et al. [35] also mentioned, and the analysis model should rather be determined based on study designs and patient characteristics. In clinical meta-analyses that combine studies involving patients with different characteristics treated across multiple institutions, the fixed effects model is rarely suitable [36]. In radiation oncology, where patients commonly have intractable diseases with diverse clinical characteristics, using the random effects model in meta-analyses might be more useful for understanding the range of effects and clinical decision-making.

In summary, meta-analyses in radiation oncology should consider heterogeneity among studies, as intractable cases of unspecified clinical features are commonly encountered. The presence of heterogeneity does not make analysis meaningless, but the clinical significance and cause of the dispersions, as well as effect size, should be discussed. The analysis model should be determined based on the designs or clinical characteristics of the included studies, and the random effects model might be considered in radiation oncology because it is difficult to conduct studies that are functionally identical or that share one common effect size.

## 4. Meta-Analyses of Observational Studies

RCTs have been known to be the best method for controlling biases within and among studies, and data obtained from meta-analyses of RCTs are considered the firmest type of evidence [1]. However, especially in the field of oncology, solving clinical questions via results based on RCTs is not always feasible. RCTs commonly require large human resources and high costs; hence, it is difficult to use them to answer all urgent health issues. An additional limitation is that the generalization of findings to patients outside the study population might not always be valid [37].

Although controversial, the number of meta-analyses of observational studies has recently been increasing [38]. The primary advantage of such studies is that they can overcome the so-called “dark matters” of clinical medicine, which are the information gaps resulting from the notion that RCT-based data provide insufficient actionable evidence [37]. Radiation oncology is one of those areas in which such dark matters exist more than in any other clinical practice.

Our recent study regarding the re-irradiation of locoregional rectal cancer recurrences [39] could be a typical example of the above. For patients with rectal cancer who have lymph node metastases or tumor invasion greater than T3, neoadjuvant chemoradiotherapy is considered standard [40]. Although locoregional recurrence after modern multidisciplinary treatments is not very common [41], no standardized treatment has been established in the event that it occurs. Repeat surgery requires good performance and the lack of comorbidities, and it is uncertain how much oncologic benefit can be obtained from performing surgery in patients with heavily treated lesions.

Physicians at multidisciplinary meetings have inquired about the application of re-irradiation. Most relevant studies have been retrospective case series, and the number of patients as well as their clinical features have varied considerably. Nevertheless, the need to evaluate the feasibility and efficacy of re-irradiation is critical for clinical decision-making. Our previous meta-analysis revealed that the two-year survival rates were 71.8% (95% CI: 54.6–84.4%) and 34.2% (95% CI: 20.4–51.2%) in patients who underwent surgery plus re-irradiation and re-irradiation alone, respectively; the rate of grade ≥3 late complications was 25.2% (range, 16.7–40%) among patients who underwent surgery, with the odds ratio of such complications being 6.4 (95% CI: 3.2–12.7) [39]. Based on these results, we can suggest that active local treatment may be effective, although candidates for surgery must be selected carefully. Although the study has several limitations, it can remain a helpful reference for actual clinical applications, at least more so than manual reading of literature that addresses vague numerals.

Previous reviews of high-quality observational studies showed effect sizes that were not significantly different from those of RCTs. Concato et al. [42] reported that the effect sizes of meta-analyses of RCTs and of high-quality cohort studies were similar. MacLehose et al. [43] also concluded that discrepancies for low-quality studies might be large while those for high-quality studies might be small. The Newcastle–Ottawa scale is one of the commonly used measures to assess the quality of observational studies in meta-analyses [44]. Studies in radiation oncology mostly use secure treatment records and evaluate objective oncologic outcomes, including overall or cause-specific survival. The representativeness of the cohorts is usually high because the evaluated clinical cases are commonly limited to a narrow range of subjects. Therefore, with some exceptions, clinical studies in radiation oncology tend to score relatively high on the Newcastle–Ottawa scale. Especially when performing meta-analyses of observational studies, possible publication bias should be evaluated and considered. The funnel-plot method [45] can show whether the results of a study are symmetrically distributed considering the sample size of each study; an asymmetrical distribution indicates possible publication bias. Fail-safe N methods [46] and Egger’s test [47] can evaluate bias quantitatively based on interpretations of funnel plots. Methods that evaluate such possible biases as well as statistical processes to adjust them, such as Duval and Tweedie’s “trim and fill” method [48] that estimates the effects of possibly missing studies, are also encouraged.

RCTs might provide the firmest evidence for use in meta-analyses with the lowest risk of possible biases. However, in radiation oncology, information gaps exist that cannot be filled by relying solely on RCT-based results. Meta-analyses of observational studies might be the best available method to fill these information gaps in order to be clinically applicable. Efforts to select high-quality studies and to reduce the possible publication biases using statistical methods are strongly encouraged.

Table 1 presents the summarized viewpoints suggested for meta-analyses in radiation oncology in regard to describing their utility, addressing heterogeneity, and incorporating observational studies.

## 5. Direction for Future Research

When analyzing qualities of systematic reviews or meta-analyses in radiation oncology [26], the median AMSTAR (Assessing the Methodological Quality of Systematic Review) score (which assesses the methodological quality of systematic reviews [49]) for systematic reviews was low (3 points). On the other hand, studies involving meta-analyses corresponded to good methodological qualities with a median of 7 points. The major cause of forgoing formal meta-analyses was heterogeneity among studies. Radiation oncology is a field where patients with intractable diseases and heterogeneous conditions commonly visit, as we mentioned above, and in which technological advances and changes in indications occur rapidly. In such a field, the presence of heterogeneity among studies is inevitable. The aim of a meta-analysis is not only to integrate RCTs to draw robust conclusions but also to provide relevant and accessible information drawn from a large pool of literature and to consequently help make clinical decisions [50]. Therefore, once again, heterogeneity among literature results might not be an obstacle for conducting formal meta-analyses; rather, they can be interpreted with clinical expertise and statistical methods such as meta-regression or subgroup comparison.

One of the strengths of radiation oncology research is that a majority of studies are conducted for academic purposes and are free from financial conflicts of interest [30]. However, the vast majority of radiation oncology meta-analyses did not document the conflicts of interest of the included studies [26]. Lane et al. [51] reported that academic meta-analyses are of a significantly higher quality than industry-supported meta-analyses. Therefore, documenting and considering the conflicts of interest of the included studies as well as of the meta-analysis itself are recommended, especially in the field of radiation oncology.

The publication statuses and assessments of publication bias within studies have not been described in more than half of the meta-analyses published on radiation oncology [26]. Whether to include unpublished materials cannot be determined uniformly. The inclusion of unpublished materials might provide more rapidly updated information within academia, provide results based on minority hypotheses where there is a lack of consensus regarding a particular subject of concern, and consequently reduce the possibility of publication bias. However, it is not uncommon for gray literature to reveal methodological flaws or report different results in subsequent full-text publications [6,7]. Therefore, the inclusion of unpublished materials should be decided after a thorough discussion between clinicians and statisticians, and the considerations taken when deciding to include them should be well described. Efforts to reduce publication biases through statistical methodologies such as funnel plots [48], fail-safe numbers [52], and the “trim and fill” method should be strongly considered. The above discussion regarding present-day problems and future directions is summarized in Table 2.

## 6. Conclusions

Meta-analyses in radiation oncology should be performed for more practical purposes and should not be limited to the conventional approach of only analyzing RCTs to obtain the highest level of evidence. Observational studies or those with heterogeneities might also be considered to estimate the clinical courses of intractable diseases and to answer detailed clinical questions that are difficult for RCTs alone to address. The quality of studies should be carefully evaluated, and the use of statistics to reduce bias is encouraged. Consideration of commonly ignored aspects such as conflicts of interests, publication status and possible biases can help increase the quality of meta-analyses related to radiation oncology. Meta-analytical results based on the expertise of radiation oncologists will be helpful for efficient clinical performance through a multidisciplinary approach.

For practical help, we summarize definitions and interpretations of selected concepts regarding meta-analysis in Table 3.

## Figures and Tables

**Table 1:** Suggested new viewpoints for meta-analyses in radiation oncology.

	Conventional Viewpoints	New Viewpoints for Radiation Oncology
Utility of meta-analysis	To build the highest level of evidence and more robust conclusions	To provide practical information serving clinical decisions for intractable diseases
Issues of heterogeneity	Avoid if possible, to draw firm and undisputed conclusions	Heterogeneity is inevitable and reflects real-life clinical situations Dispersion of effects sizes are subjects of clinical discussion
Analysis of observational studies	Prefer to analyze only RCTs, reducing biases and heterogeneity among studies	Important to fill the gaps between RCT results and clinical decisions Efforts to select high-quality studies and reduce biases are encouraged

RCT, randomized controlled trial.

**Table 2:** Summary of directions for future research.

Problems Identified	Future Directions
Low methodological quality (low score without formal meta-analysis)	Conducting a formal meta-analysis despite heterogeneities Interpret heterogeneities based on a combination of clinical expertise and statistical methods (e.g., subgroup comparison or meta-regression, sensitivity analysis) rather than avoid such interpretations and limit discussion to narrative descriptions
Scarce information of CoI (vast majority of meta-analyses did not document CoIs in the included studies)	Document CoI of studies included in meta-analyses and discuss as relevant, because academic studies might have better qualities than studies with industrial CoI (majority of radiation oncology studies have a merit of being free from CoI)
Lack of consideration for study inclusion according to publication status or publication bias	Inclusion of unpublished materials could not be uniformly suggested Thorough discussions by clinical and statistical experts are essential, including the addressing of methods to reduce possible biases

CoI, conflicts of interests.

**Table 3:** Selected concepts in meta-analysis [17,53,54,55,56].

Category	Concept	Definition	Common Usages or Interpretation
Effects models for pooled analyses	Fixed effects model	A model based on the assumption that all studies in the analysis are functionally identical and that there is a common effect size	For RCTs with very similar design; repetitive lab study samples
	Random effects model	A model assumes that the true effect size varies among studies and is used to estimate the mean distribution of the effects	For studies from different institutions, meta-analysis including observational studies
Heterogeneity analysis	Cochran’s Q test	The test of null hypothesis Q that all studies have a common effect size	Commonly interpreted in practice as, that to reject Q if *p*-value < 0.1; I^2^ interpretation (Higgins et al. [57].): 25%, 50%, and 75% denote borderlines of low, moderate, and high heterogeneities. However, the values should not be interpreted only in a categorical way but also clinically and quantitatively.
	I^2^ value (%)	A concept reflecting the proportion of variance between studies divided by total variance	
Analysis of heterogeneity	Subgroup analysis	Comparison among included study subgroups categorized by its characteristics, regarding effect sizes	*z*-test (same logic as *t*-test between two groups); analysis of variance (Q test to partition the variance and test the proportion of between-subgroups variance divided by within-studies variance)
	Meta-regression	Quantitative regression analysis using effect size as dependent variable and moderator of included studies’ characteristics as independent variable (Similar to regression analysis of primary studies)	Useful to identify dose–response relationship
	Sensitivity analysis	Analysis of whether the findings robust to the decision made in the process of obtaining them; for example, analysis with outliers and analysis without outliers	Robustness of clinical and methodological decision making (e.g., analysis with only RCT among included studies vs. analysis with RCT + observational studies)
Publication bias	Publication bias	The bias whereby statistically significant results are more likely to be published than null or non-significant results	
	Funnel plot	A scatterplot of the effect estimates from studies included against some measure of size or precision of each study (powerful studies locate toward top of the plot shaped as a reversed funnel, while smaller studies scatter more widely at the bottom)	Visually inspected asymmetry suggests possible publication bias. Quantitative statistical methods, such as Egger’s test, yield *p*-value, which is more familiar to clinicians. Trim and fill methods can estimate adjusted effect size considering publication biases from missing studies.

RCT, randomized controlled trial.

## Data Availability

All data generated or analysed during this study are included in this published article.
